# Functional Assessment of Four Novel Immune-Related Biomarkers in the Pathogenesis of Clear Cell Renal Cell Carcinoma

**DOI:** 10.3389/fcell.2021.621618

**Published:** 2021-03-16

**Authors:** Daojun Lv, Xiangkun Wu, Ming Wang, Wenzhe Chen, Shuxin Yang, Yongda Liu, Guohua Zeng, Di Gu

**Affiliations:** ^1^Department of Urology, Minimally Invasive Surgery Center, The First Affiliated Hospital of Guangzhou Medical University, Guangzhou, China; ^2^Guangdong Key Laboratory of Urology, Guangzhou Institute of Urology, Guangzhou, China

**Keywords:** clear cell renal cell carcinoma, immune-related biomarkers, diagnosis and prognosis, robust rank aggregation, weighted gene co-expression network analysis, tumor microenvironment

## Abstract

**Background:**

Clear cell renal cell carcinoma (ccRCC) is the most common subtype of renal cell carcinoma whose pathogenesis is not well understood. We aimed at identifying novel immune-related biomarkers that could be valuable in the diagnosis and prognosis of ccRCC.

**Methods:**

The Robust Rank Aggregation (RRA) method was used to integrate differently expressed genes (DEGs) of 7 Gene Expression Omnibus (GEO) datasets and obtain robust DEGs. Weighted gene co-expression network analyses (WGCNA) were performed to identify hub genes associated with clinical traits in The Cancer Genome Atlas (TCGA) database. Comprehensive bioinformatic analyses were used to explore the role of hub genes in ccRCC.

**Results:**

Four hub genes IFI16, LMNB1, RHBDF2 and TACC3 were screened by the RRA method and WGCNA. These genes were found to be up-regulated in ccRCC, an upregulation that could be due to their associations with late TNM stages and tumor grades. The Receiver Operating Characteristic (ROC) curve and Kaplan-Meier survival analysis showed that the four hub genes had great diagnostic and prognostic values for ccRCC, while Gene Set Enrichment Analysis (GSEA) showed that they were involved in immune signaling pathways. They were also found to be closely associated with multiple tumor-infiltrating lymphocytes and critical immune checkpoint expressions. The results of Quantitative Real-time PCR (qRT-PCR) and immunohistochemical staining (IHC) analysis were consistent with bioinformatics analysis results.

**Conclusion:**

The four hub genes were shown to have great diagnostic and prognostic values and played key roles in the tumor microenvironment of ccRCC.

## Introduction

Renal cell carcinoma (RCC) is the third most common malignancy of the urinary system, accounting for approximately 3% of all malignancies (Siegel et al., 2020). According to the global cancer statistics (2018), there were about 403,262 (2.2%) new cases of RCC with a mortality rate of 1.8% (Bray et al., 2018). Clear cell renal cell carcinoma (ccRCC), accounting for over 80% of all RCC cases, is the most common pathological subtype of RCC (Hsieh et al., 2017). An estimated 30% of all ccRCC cases are diagnosed in the metastatic stages with recrudescence occurring in 20 to 30% of patients who have undergone partial or radical nephrectomy (Cairns, 2010; Wolff et al., 2016; Hsieh et al., 2017). Targeted therapies such as sunitinib (Motzer et al., 2006), sorafenib (Hutson et al., 2010) and axitinib (Hutson et al., 2013) are important as first-line ccRCC medications. These therapies, coupled with immunotherapy have a positive prognostic outcome in ccRCC patients (Rini et al., 2019). Despite the advances in the therapeutic management of ccRCC, the recovery rate of these patients is still low (Vera-Badillo et al., 2015; Tsimafeyeu et al., 2017). Therefore, identify more novel immune-related biomarkers that could be vital in the diagnosis, treatment and prognosis of ccRCC is urgent.

The development of high-throughput technologies and bioinformatic advancements have led to the identification of novel ccRCC biomarkers (Mitchell et al., 2018; Linehan and Ricketts, 2019). However, research using small sample sizes and different sequencing platforms has resulted in great variabilities and poor statistical inferences among studies. The Robust Rank Aggregation (RRA) method can integrate differentially expressed gene (DEG) lists of different datasets, thereby overcoming the challenges posed by small sample sizes (Kolde et al., 2012). In addition, the use of DEGs between different samples, while ignoring the internal relationship between genes, can help overcome this problem. Genes with extremely similar expression patterns in different samples can be identified by the weighted gene co-expression network analysis (WGCNA) (Langfelder and Horvath, 2008). This analysis screens out biomarkers based on internal relationships among genes and correlates gene sets with their clinical traits (Langfelder and Horvath, 2008).

In this study, we aimed at identifying novel immune-related biomarkers that were significantly associated with the progression and prognosis of ccRCC. Moreover, we investigated the potential molecular mechanisms of these biomarkers, as well as their associations with the tumor microenvironment (TME). Finally, we performed Quantitative Real-Time PCR (qRT-PCR) analysis to detect the expression of novel immune-related biomarkers in clinical ccRCC samples.

## Materials and Methods

### Data Source and Preprocessing

**FIGURE 1:**
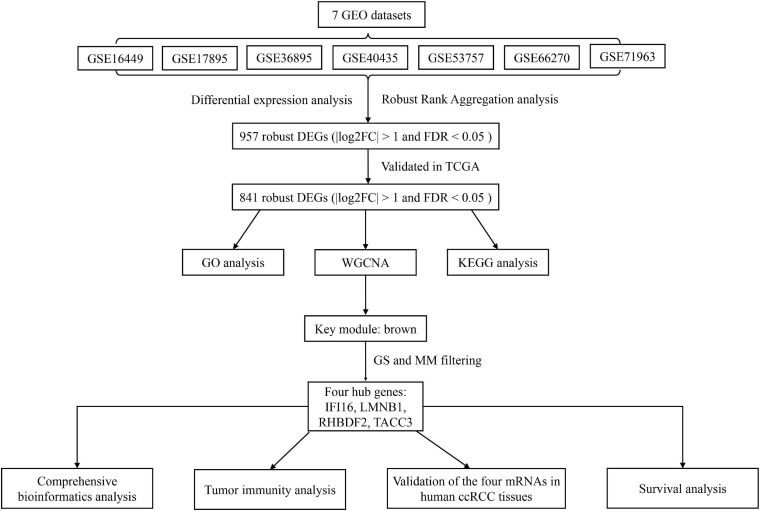
A workflow showing the process of screening out hub genes and their comprehensive analysis.

The workflow of this study is shown in Figure 1. The matrix files of eligible microarray datasets were obtained from the Gene Expression Omnibus (GEO) database^1^. Datasets that had human renal tissue samples and contained at least 10 tumor- and non-tumor renal control tissue samples were included in this study. Cell line and xenograft tissues were excluded from this study. Accordingly, 7 GEO datasets were included for DEG analysis, including GSE71963, GSE66270, GSE53757, GSE40435, GSE36895, GSE17895, and GSE16449. The probes were matched to the gene symbols using the annotation files of the respective platforms. Normalized RNA-sequencing data as FPKM (Fragments Per Kilobase Million) and the corresponding pathological information of ccRCC samples were downloaded from The Cancer Genome Atlas (TCGA) database^2^. In total, 517 TCGA-ccRCC and 89 CPTAC-ccRCC samples that had complete clinic-pathological data with follow up time were included into this study.

### Identification of Robust DEGs

DEGs between the adjacent normal tissue and ccRCC samples were identified by the ‘limma’ package (version 3.44.3^3^), while DEG integration of the 7 microarray datasets to obtain robust DEGs was achieved using the RRA method. In the RRA analysis, | log2-fold change (FC)| and false discovery rate (FDR) < 0.05 were set as the cutoff points for robust DEGs based on the ‘RobustRankAggreg’ package (version 1.1^4^). Robust DEGs were further validated between paired ccRCC and adjacent samples in the TCGA-ccRCC database using the ‘edgeR’ package (version 3.30.0^5^). Statistical significance was set at | log2FC| > 1 and FDR < 0.05.

### Function Enrichment Analysis of Robust DEGs

Gene Ontology (GO) enrichment, including molecular functions (MF), cellular components (CC) and biological processes (BP), and the Kyoto Encyclopedia of Genes and Genome (KEGG) pathway analyses were conducted with the ‘clusterprofiler’ package (version 3.16.1^6^). GO terms or KEGG pathways with FDR < 0.05 were visualized by the “GOplot” package.

### WGCNA and Identification of the Key Module

The expression data of robust DEGs were retrieved from the TCGA database and used in the WGCNA analysis. The WGCNA method was important in constructing co-expression networks and identifying clinical traits related to DEGs. Pearson’s correlations between all pair-wise genes were used to generate the adjacency matrix, while the soft threshold power of β = 5 was used to achieve scale-free topology of the adjacency matrix. The adjacency matrix was then transformed into a topological overlap matrix (TOM). This transformation was done based on the TOM-based dissimilarity measure with a minimum module size of 30 and cut height of 0.25. Robust DEGs with similar expression patterns were classified into the same gene module by average linkage hierarchical clustering. The correlation between module eigengenes (MEs) and clinical traits was calculated to identify clinically significant modules. Finally, robust DEGs with a gene significance (GS) > 0.3 and a module membership (MM) > 0.7 were selected as hub genes.

### Comprehensive Bioinformatic Analyses of Hub Genes

The TIMER website^7^ was used to validate the differences in hub gene expression between pan-cancer and adjacent normal tissues. To identify the diagnostic value of these hub genes in TCGA ccRCC, Receiver Operating Characteristic (ROC) curve analysis was performed and the area under the curve (AUC) was calculated using the “pROC” package (version 1.10.0^8^). The ‘ggstatsplot’ package (version 0.6.1^9^) was used to assess hub gene expression between different T stages, AJCC stages and tumor grades.

Normal tissue protein level and ccRCC data were obtained from the CPTAC database^10^ and used to identify the protein level of the hub genes. Moreover, ROC curve analysis was performed to assess the diagnostic value of these proteins.

Exploration of enriched KEGG pathways of the hub genes was achieved using the Gene Set Enrichment Analysis (GSEA) 4.0.1 software. Based on each hub gene’s median expression, the 517 ccRCC samples were divided into high- and low-expression groups. “c2.cp.kegg.v7.1.symbols.gmt” as the reference gene set was acquired from the Molecular Signatures Database V7.1 (MSigDB). Statistical significance was set at FDR < 0.05 and | Normalized Enrichment Score (NES)| > 1.

### Tumor Immunity Analysis of Hub Genes

The estimate, stromal and immune scores of each TCGA ccRCC sample were downloaded from the ESTIMATE bioinformatics website^11^ and used to determine the association between hub genes and tumor purity and the association between the infiltration level of immune cells and the level of stromal cells in ccRCC tissues. The ESTIMATE algorithm is based on a single sample GSEA to evaluate tumor purity.

The TIMER website^7^ was used to explore the relationships between hub gene expression and abundance of tumor-infiltrating lymphocytes (TILs) such as CD8 + T cells, CD4 + T cells, B cells, dendritic cells, macrophages and neutrophils (Li et al., 2017). Estimation of 22 TIL compositions from bulk tissues based on their gene expression profiles was performed using the CIBERSORT method (Chen et al., 2018). The LM22 signature matrix was used to identify the 22 TILs containing seven T cell types, natural killer (NK) cells, myeloid subsets, plasma cells and naive and memory B cells. To further identify the relationships between hub genes and TILs, the CIBERSORT website^12^ in combination with the FPKM data of TCGA ccRCC and the LM22 signature matrix was used to estimate the TIL fractions. The sum of the 22 estimated TIL fractions in each sample is equal to 1. Spearman rank correlation analysis was used to evaluate the relationships between TILs. Moreover, the TISIDB website^13^ was used to explore the associations between hub genes and critical immune checkpoint inhibitors (ICIs: CTLA4, HAVCR2, LAG3, PDCD1, and TIGIT).

### Survival Analysis of Hub Genes

A total of 517 TCGA-ccRCC samples were divided into high- and low- expression groups based on best cutoff points calculated by the ‘survminer’ package (version 0.4.8^14^). The Kaplan-Meier survival analysis with the log-rank test was then conducted to identify prognosis-related genes using the “survival” package (version: 3.2-7^15^). Furthermore, 89 CPTAC-ccRCC samples were used to validated the prognosis of the hub genes. To validate whether hub genes were risk factors independent of clinical-pathological variables (age, T stage, N stage, M stage, AJCC stage and tumor grade) in ccRCC patients, univariate and multivariate Cox proportional hazards regression analyses were performed.

### Cell Lines and Culture

Human normal kidney cell line HK-2 and ccRCC cell lines 786-O, OSRC-2, Caki-1, SN12-PM6 and SW839 were purchased from the American Type Culture Collection (ATCC, Manassas, VA, United States). The cells were cultured in 1640 Medium (Invitrogen, Grand Island, NY, United States) supplemented with 10% FBS (GIBCO, Brazil), penicillin (25 units/ml), streptomycin (25 g/ml), and 1% L-glutamine at 37°C with 5% CO2.

### RNA Extraction and qRT-PCR

Total RNA was isolated with TRIzol reagent (TaKaRa Biomedical Technology, Dalian, China) according to the manufacturer’s instructions. Complementary DNA was reverse-transcribed using the Prime Script RT reagent Kit (TaKaRa). the qRT-PCR analysis was conducted using TB^®^ Green PCR Master Mix (TaKaRa). The specific primers set for IFI16, LMNB1, RHBDF2, TACC3, CD4, CD8 and GAPDH are presented in Supplementary Table 1. All data analyses were managed using RocheLightCycler480. Data were calculated from three biological and technical replicates then normalized to GAPDH expression levels using the 2^–ΔΔ*Ct*^ method.

### Patients and Clinical Samples

Primary ccRCC patients who underwent radical surgery without any preoperative chemotherapy or radiotherapy at the First Affiliated Hospital of Guangzhou Medical University between 2016 and 2019 were enrolled in the study. As for Formalin fixed paraffin—embedded ccRCC specimens, a total of 150 patients diagnosed with primary ccRCC who underwent operation at the Department of Urology of the First Affiliated Hospital of Guangzhou Medical University (Guangzhou, China) and Nanfang hospital (Guangzhou, China) between February 2008 to August 2015 were enrolled in our study. The follow up of participants (*n* = 150) were gotten through phone calls until death or the cut-off date of August 1, 2015. The mean follow-up time was 68 months (from 4.0 to 90.0 months). All the deaths were ascribed to ccRCC. Pathological TNM staging was reassessed in accordance with the American Joint Committee on Cancer (AJCC). Histological and pathological diagnoses of the specimens was assigned basing on the 2007 World Health Organization (WHO) Consensus Classification and Staging System of Renal Tumor and Fuhrman grade by two experienced pathologists. Written informed consent was obtained for all patients before specimen collection, following the ethical protocols of the Ethics Committee of the First Affiliated Hospital of Guangzhou Medical University. All the study protocol was approved by the Ethics Committee of the First Affiliated Hospital of Guangzhou Medical University and Southern Medical University Institutional Board.

### Tissue Microarray Construction and Immunohistochemistry (IHC)

Tissue microarray (TMA) was established from 150 formalin-fixed paraffin-embedded human ccRCC tissues block according to the standard methods. IFI16, RHBDF2, TACC3 protein expression was confirmed using an immunoperoxidase method. The tissue array was deparaffinized, rehydrated, and inhibited endogenous peroxidase activities by 0.3% H2O2 for 30 min. For antigen retrieval, the slides were boiled in sodium citrate buffer (10 mM, pH 6.0) in a pressure cooker for 7 min. Afterward, non-specific binding was blocked with 5% normal goat serum, and then incubated with primary antibody (IFI16, 1:200, ab13454, Abcam Inc., Cambridge, MA, United States). (RHBDF2, 1:500, abs10947, Absin, Shanghai, China), (TACC3, 1:100, 14970s, Cell Signaling Technology, Inc., United States). Sequentially tissue array was incubated with polyperoxidase-anti-mouse IgG (Zhongshan Biotech. China). Diaminobenzidine (DAB) was visualized as a chromogen substrate. The nucleus was counterstained with hematoxylin. IFI16, RHBDF2, TACC3 staining in nuclear was reckoned as detectable immunoreactions. To evaluate the consequences of IFI16, RHBDF2, TACC3 staining, the intensity and percentage of cells in cancerous and non-cancerous tissues were scored by two pathologists independently. The intensity of staining was determined in accordance with the following scale: 0 (no staining); 1 (weak staining, light yellow); 2 (moderate staining, yellowish brown) and 3 (strong staining, brown). Based on the percentage of positively stained tumor cells, the score of staining extent was denoted on 4point scale as follows: 0 (less than 5%); 1 (5 to 25%); 2 (25 to 50%); 3 (more than 75%). The final scores were then calculated according to score × proportion of positive tumor cells for IFI16, RHBDF2, TACC3 expression (range from 0 to 9). Tumor tissues with scores of 0–1 was recognized as low expression because approximately 90% of normal kidney tissues expressed a low level of IFI16, RHBDF2, TACC3 with an IHC score of ≤ 1 in our preliminary study. Then we defined 0-1 as low expression and 2–9 as high expression.

### Statistical Analysis

The hazard ratio (HR) and 95% confidence interval (CI) were calculated by Cox regression analysis and Kaplan-Meier survival analysis. Spearman correlation analysis was used to evaluate the correlation between two continuous variables. The Kruskal-Wallis or student’s t-test was used to compare between groups for continuous variables. When multiple comparisons were performed, *p*-values were corrected according to the FDR method. The FDR method was used to control for multiple testing that could lead to a false positive. All experiments were repeated thrice and the data were presented as means and standard deviation (SD) in all plots shown in this study unless differently stated in the legend. All statistical analyses were conducted by R software (version 3.6.2^16^) and all *P* < 0.05 (2-sided) were considered statistically significant.

## Results

### Identification of Robust DEGs

Details of 7 eligible GEO datasets (GSE71963, GSE66270, GSE53757, GSE40435, GSE36895, GSE17895 and GSE16449) are shown in Table 1 with their DEG identifiers (5314, 3180, 4033, 7082, 4090, 6250, and 5334 DEGs, respectively). A total of 957 robust DEGs in GEO datasets were detected by RRA analysis. Among these, 841 DEGs with downregulated (454 DEGs) and up-regulated (387 DEGs) mRNAs were validated between paired ccRCC and adjacent samples in a TCGA-ccRCC database (Supplementary Figure 1A). The top 20 up- and down-regulated robust DEGs detected using RRA analysis are listed in Supplementary Figure 1B.

**TABLE 1:** Details of 7 GEO datasets included in this study.

Dataset ID	Sample size		Platform
	Normal	Tumor	
GSE71963	16	32	Agilent-014850 Whole Human Genome Microarray 4 × 44K G4112F
GSE66270	14	14	[HG-U133_Plus_2] Affymetrix Human Genome U133 Plus 2.0 Array
GSE53757	72	72	[HG-U133_Plus_2] Affymetrix Human Genome U133 Plus 2.0 Array
GSE40435	101	101	Illumina HumanHT-12 V4.0 expression beadchip
GSE36895	23	53	[HG-U133_Plus_2] Affymetrix Human Genome U133 Plus 2.0 Array
GSE17895	22	138	Affymetrix GeneChip Human Genome U133 Plus 2.0 Array (MBNI v11 Entrez Gene ID CDF)
GSE16449	52	18	Agilent-014850 Whole Human Genome Microarray 4 × 44K G4112F

### Functional Enrichment Analysis of Robust DEGs

**FIGURE 2:**
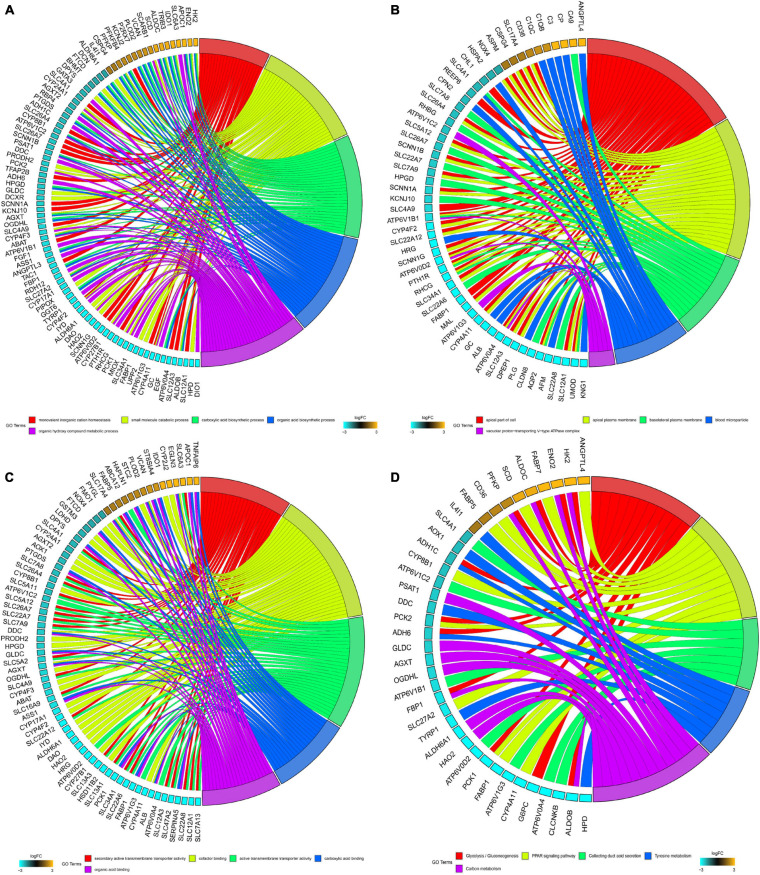
GO and KEGG analysis of robust DEGs. **(A)** Chord plot shows the relationship between genes and GO terms of biological process. **(B)** Chord plot shows the relationship between genes and GO terms of cellular component. **(C)** Chord plot shows the relationship between genes and GO terms of molecular function. **(D)** Chord plot shows the relationship between genes and KEGG pathways (GO, Gene Ontology; KEGG, Kyoto Encyclopedia of Genes and Genomes).

Significantly enriched BP of robust DEGs was identified, including monovalent inorganic cation homeostasis, small molecule catabolic process, carboxylic acid biosynthetic process and organic acid biosynthetic process (Figure 2A). Several CC GO terms were detected, including apical part of the cell, apical plasma membrane, basolateral plasma membrane and blood microparticle (Figure 2B). In GO terms of MF, secondary active transmembrane transporter activity, active transmembrane transporter activity and cofactor binding were significantly enriched terms (Figure 2C). Based on KEGG pathway analysis, glycolysis/gluconeogenesis, PPAR signaling pathway and collecting duct acid secretion were mostly associated with the robust DEGs (Figure 2D).

### WGCNA and Identification of Key Modules and Hub Genes

**FIGURE 3:**
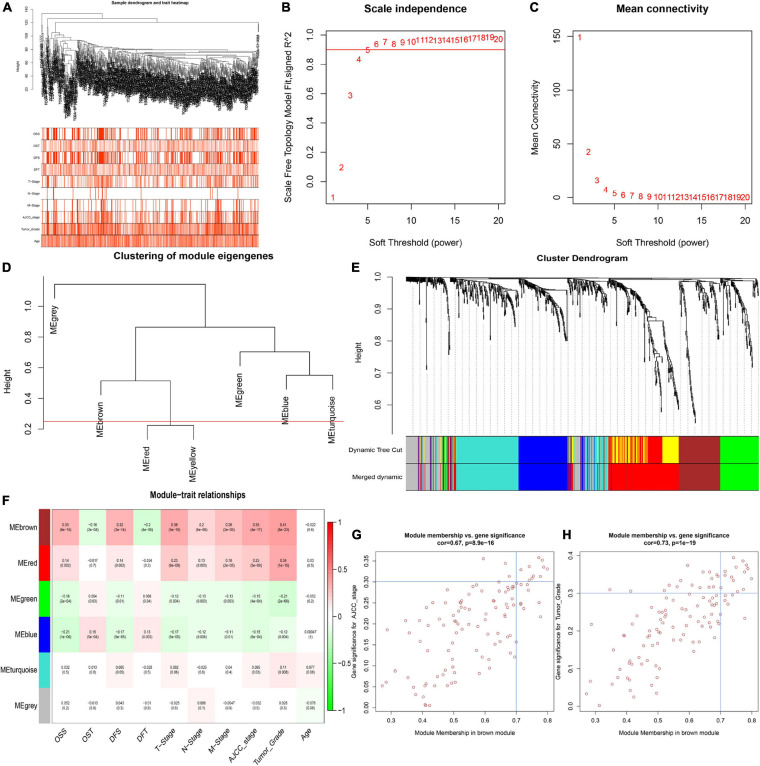
Identification of key modules correlated with clinical traits in the TCGA-ccRCC dataset. **(A)** Clustering dendrograms of robust DEGs. Color intensity varies positively with age, overall survival time (OST), disease free time (DFT), T stage, N stage, M stage, AJCC stage and tumor grade. In terms of overall survival status (OSS) and disease-free status (DFS), red means dead or disease progression and white indicates live or disease free. **(B–C)** Analysis of scale-free fit index **(B)** and mean connectivity **(C)** for various soft-thresholding powers. **(D)** Clustering of module eigengenes. The red line shows cut height (0.25). **(E)** Dendrogram of robust DEGs clustered based on a dissimilarity measure (1- TOM). **(F)** Heatmap of the correlation between module eigengenes and clinical traits of ccRCC. Each cell contains *p*-value and the correlation coefficient. **(G)** Scatter plot of module eigengenes related to AJCC stage in the brown module. **(H)** Scatter plot of module eigengenes related to tumor Grade in the brown module.

To identify key modules significantly related to ccRCC clinical traits, WGCNA was performed on the TCGA-ccRCC dataset incorporating 841 robust DEGs derived from the previous analysis (Figure 3). Clinical information of ccRCC patients such as age, overall survival status (OSS), overall survival time (OST), disease-free status (DFS), disease-free time (DFT), T stage, N stage, M stage, AJCC stage and tumor grade were retrieved from TCGA (Figure 3A). By setting the cut height at 0.25 and β at 5 (scale-free R2 = 0.85), 841 robust DEGs were divided into six modules (Figures 3B–E). As shown in the heatmap of the module-trait relationship, the brown module was significantly related to clinical traits (Figure 3F). Gene significance > 0.3 of AJCC stage and tumor grade and MM > 0.7 were selected as cutoff points (Figures 3G,H). We identified 4 hub genes from the brown module: IFI16, LMNB1, RHBDF2 and TACC3.

### Comprehensive Bioinformatic Analyses of Hub Genes

**FIGURE 4:**
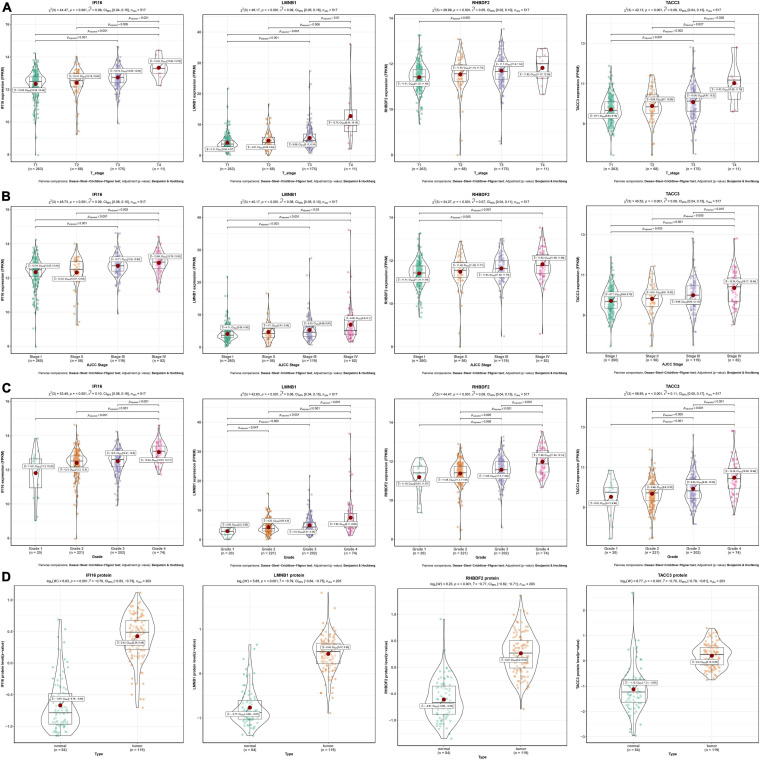
Validation of four hub genes in the TCGA-ccRCC database. **(A)** Expression of IFI16, LMNB1, RHBDF2 and TACC3 in ccRCC samples with different T stages. **(B)** Expression of IFI16, LMNB1, RHBDF2 and TACC3 in ccRCC samples with different AJCC stages. **(C)** Expression of IFI16, LMNB1, RHBDF2 and TACC3 in ccRCC samples with different tumor grades. **(D)** Corresponding protein levels between ccRCC and adjacent normal tissue.

As shown in the pan-cancer view (Supplementary Figure 2), the 4 hub genes (IFI16, LMNB1, RHBDF2, and TACC3) were significantly up-regulated in ccRCC samples and other cancer types when compared to adjacent normal tissues (*p* < 0.001). Furthermore, the ROC curve analysis showed that these hub genes had a high diagnostic value as biomarkers for TCGA ccRCC (IFI16 AUC: 0.921, LMNB1 AUC: 0.87, RHBDF2 AUC: 0.957, TACC3 AUC: 0.896; Supplementary Figure 3A). These genes were significantly differentially expressed in ccRCC samples with different T stages, AJCC stages and tumor grades (*p* < 0.001). Higher expression levels were an indication of advanced T stages, AJCC stages and tumor grades (Figures 4A–C).

The protein levels of the 4 hub genes were significantly up-regulated in ccRCC samples compared to normal tissues (*p* < 0.001) (Figure 4D). The ROC curve analysis revealed that these genes had good efficacies in the diagnosis of ccRCC (IFI16 protein AUC: 0.955, LMNB1 protein AUC: 0.959, RHBDF2 protein AUC: 0.94, TACC3 protein AUC: 0.915; Supplementary Figure 3B).

**FIGURE 5:**
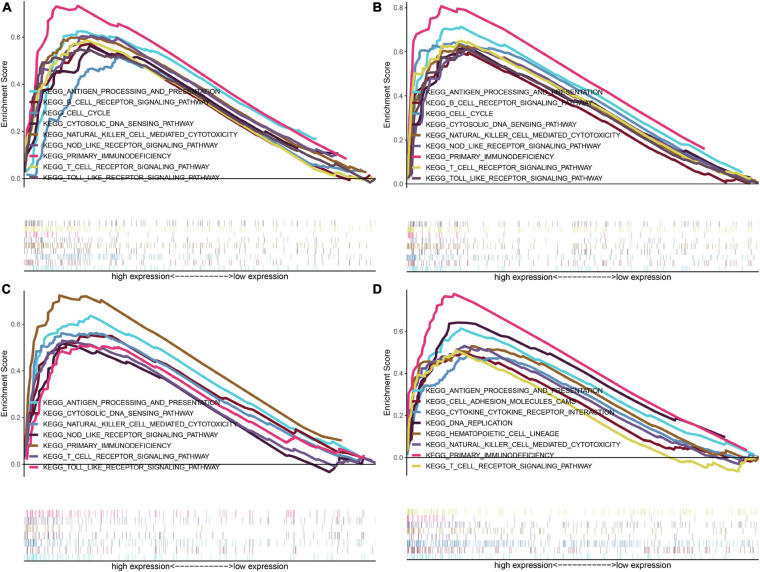
GSEA of KEGG pathway gene sets in IFI16 **(A)**, LMNB1 **(B)**, RHBDF2 **(C)**, and TACC3 **(D)** high versus low samples from TCGA database. The normalized enrichment scores (NES) are shown in each plot.

Further investigations of enriched KEGG pathways of IFI16, LMNB1, RHBDF2 and TACC3 in ccRCC showed that highly expressed genes [IFI16 (A), LMNB1 (B), RHBDF2 (C), and TACC3 (D)] were all enriched in T cell receptor signaling pathway, NK cell-mediated cytotoxicity, antigen processing and presentation and primary immunodeficiency. Meanwhile, the NOD-like receptor signaling, cytosolic DNA sensing and Toll-like receptor signaling pathways were enriched in the high-expression groups of IFI16, LMNB1 and RHBDF2. The cell cycle and B cell receptor signaling pathway were enriched in LMNB1 and IFI16 high-expression groups, respectively. The GSEA showed that LMNB1, IFI16, RHBDF2 and TACC3 were closely associated with immune signaling pathways (Figures 5A–D).

### Tumor Immunity Analysis of Hub Genes

**FIGURE 6:**
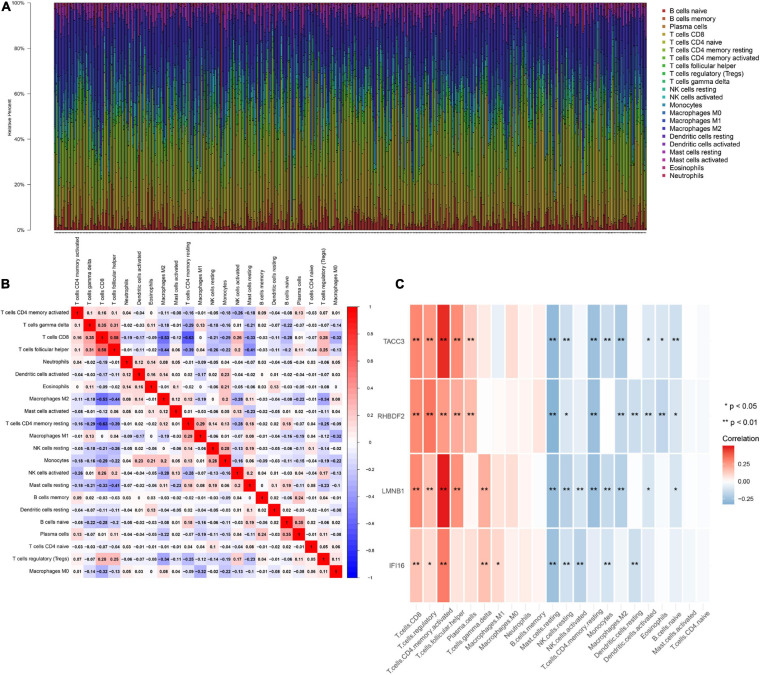
The landscape of immune infiltration in TCGA ccRCC patients. **(A)** The abundance fraction of 22 tumor-infiltrating lymphocytes (TILs) in the 396 ccRCC samples. Each column represents a sample, and each column with a different color and height indicates the abundance fraction of TILs in that sample. **(B)** The correlation between the abundance fractions of various immune cells. The value represents the correlation value. Red represents a positive correlation, and blue represents a negative correlation. **(C)** The relationship between expression of IFI16, LMNB1, RHBDF2 and TACC3 and various TILs.

**FIGURE 7:**
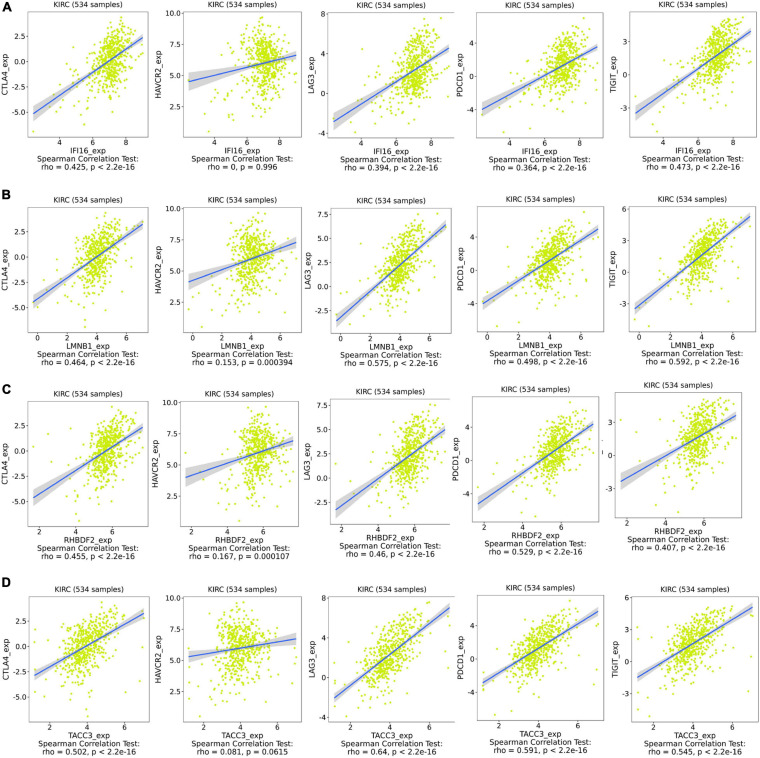
Associations between the expression of IFI16 **(A)**, LMNB1 **(B)**, RHBDF2 **(C)**, and TACC3 **(D)** with the expression of CTLA4, HAVCR2, LAG3, PDCD1, and TIGIT in ccRCC.

IFI16, LMNB1, RHBDF2 and TACC3 were positively correlated with the estimate, stromal, and immune scores (Supplementary Figure 4). These results suggest that the 4 hub genes were negatively correlated with the tumor purity of ccRCC and were up-regulated in the TME. The expression levels of IFI16, LMNB1, RHBDF2 and TACC3 positively correlated with infiltration levels of the six TILs, including CD8 + T cells, CD4 + T cells, B cells, dendritic cells, macrophages and neutrophils (Supplementary Figure 5). This shows that these genes play a key role in the immune infiltration of ccRCC. As shown in Figure 6A, abundant fractions of 22 TILs were different in each ccRCC sample. This explains the tumor heterogeneity among different individuals. In addition, different TIL subpopulation ratios were weakly to moderately correlated (Figure 6B). The analysis showed that these genes were positively correlated with multiple TILs, especially activated CD4 + memory T cells, CD8 + T cells, regulatory T cells (Treg) and follicular helper T (Tfh) cells, but were negatively correlated with resting mast cells, resting NK cells and activated NK cells (Figure 6C). There was also a positive correlation between the 4 hub genes and the expression levels of TIGIT, HAVCR2, CTLA4, PDCD1 and LAG3 in ccRCC, revealing that these genes might be associated with the immunosuppressive microenvironment (Figure 7).

### Survival Analysis of Hub Genes

**FIGURE 8:**
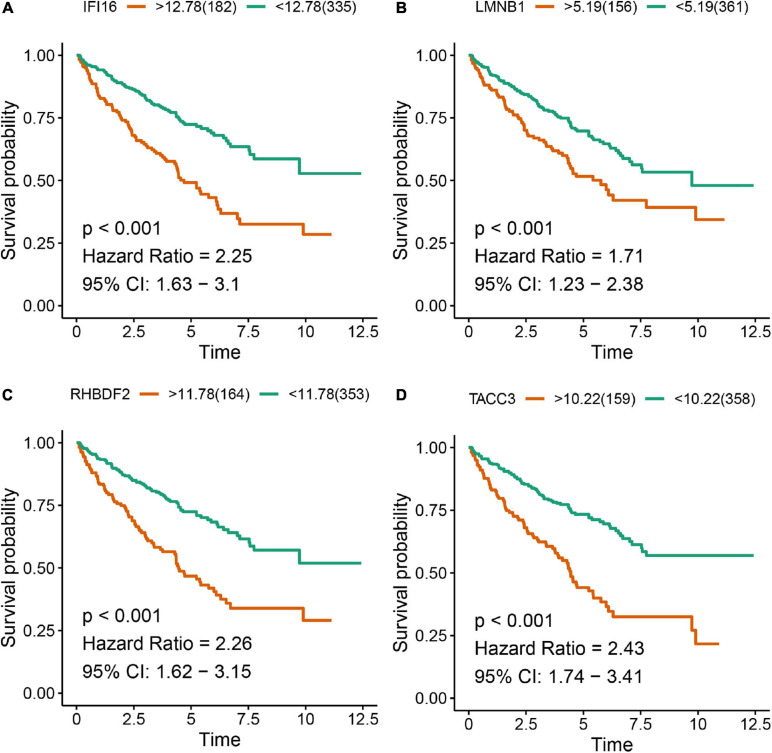
Kaplan-Meier survival analysis showing the higher expressions of IFI16 **(A)**, LMNB1 **(B)**, RHBDF2 **(C)**, and TACC3 **(D)** that were correlated with poor survival of ccRCC patients in the TCGA database.

The Kaplan-Meier survival analysis showed that higher expressions of IFI16 (*p* < 0.001, HR = 2.25, 95% CI: 1.63-3.1), LMNB1 (*p* < 0.001, HR = 1.71, 95% CI: 1.23-2.38), RHBDF2 (*p* < 0.001, HR = 2.26, 95% CI: 1.62-3.15) and TACC3 (*p* < 0.001, HR = 2.43, 95% CI: 1.74-3.41) predicted poor OS (Figures 8A–D), which were consistent with the Kaplan-Meier survival analysis results of CPTAC-ccRCC cohort (Supplementary Figure S6). In the univariate Cox proportional hazards regression analysis, 11 variables, included T stage, M stage, N stage, age, tumor grade and AJCC stage, showed statistical significance with hub gene expression. Based on the multivariate Cox proportional hazards regression analysis, the 4 hub genes were regarded as independent prognostic factors for ccRCC (Table 2).

**TABLE 2:** Cox regression analysis of four hub genes and clinical data of ccRCC in TCGA database.

Variables	Univariate analysis			Multivariate analysis 1		
	HR (95% CI)		*P*	HR (95% CI)		*P*
Age	1.02(1.01−1.04)		< 0.001	1.03(1.01−1.04)		< 0.001
T Stage (T3 and T4/T1 and T2)	3.11(2.28−4.21)		< 0.001	0.83(0.45−1.52)		0.56
N Stage (N1/N0)	3.97(2.14−7.33)		< 0.001	2.23(1.18−4.19)		0.01
M Stage (M1/M0)	4.45(3.26−6.08)		< 0.001	2.50(1.71−3.65)		< 0.001
AJCC Stage (III and IV/I and II)	3.75(2.72−5.14)		< 0.001	2.14(1.08−4.21)		0.03
Grade (G3 and G4/G1 and G2)	2.64(1.87−3.70)		< 0.001	1.58(1.10−2.29)		0.01
IFI16 expression	1.96(1.53−2.51)		< 0.001	1.63(1.26−2.11)		< 0.001
LMNB1 expression	1.90(1.60−2.25)		< 0.001		
RHBDF2 expression	2.05(1.67−2.49)		< 0.001		
TACC3 expression	2.03(1.72−2.37)		< 0.001		

**Variables**	**Multivariate analysis 2**		**Multivariate analysis 3**		**Multivariate analysis 4**	
	**HR (95% CI)**	***P***	**HR (95% CI)**	***P***	**HR (95% CI)**	***P***

Age	1.03(1.01−1.04)	<0.001	1.03(1.01−1.04)	<0.001	1.03(1.01−1.04)	<0.001
T Stage (T3 and T4/T1 and T2)	0.89(0.49−1.62)	0.72	0.89(0.48−1.62)	0.7	0.91(0.49−1.65)	0.76
N Stage (N1/N0)	1.96(1.02−3.72)	0.041	1.70(0.92−3.41)	0.08	1.65(0.86−3.15)	0.13
M Stage (M1/M0)	2.30(1.57−3.37)	<0.001	2.39(1.63−3.49)	<0.001	2.37(1.62−3.45)	<0.001
AJCC Stage (III and IV/I and II)	2.05(1.03−4.06)	0.04	2.06(1.03−4.10)	0.039	1.97(0.99−3.92)	0.051
Grade (G3 and G4/G1 and G2)	1.48(1.01−2.14)	0.04	1.48(1.02−2.15)	0.035	1.41(0.96−2.04)	0.07
LMNB1 expression	1.48(1.22−1.79)	<0.001				
RHBDF2 expression			1.51(1.21−1.86)	<0.001		
TACC3 expression					1.66(1.39−1.97)	<0.001

### Validation of the Expression of Hub Genes in Clinical ccRCC Specimens

**FIGURE 9:**
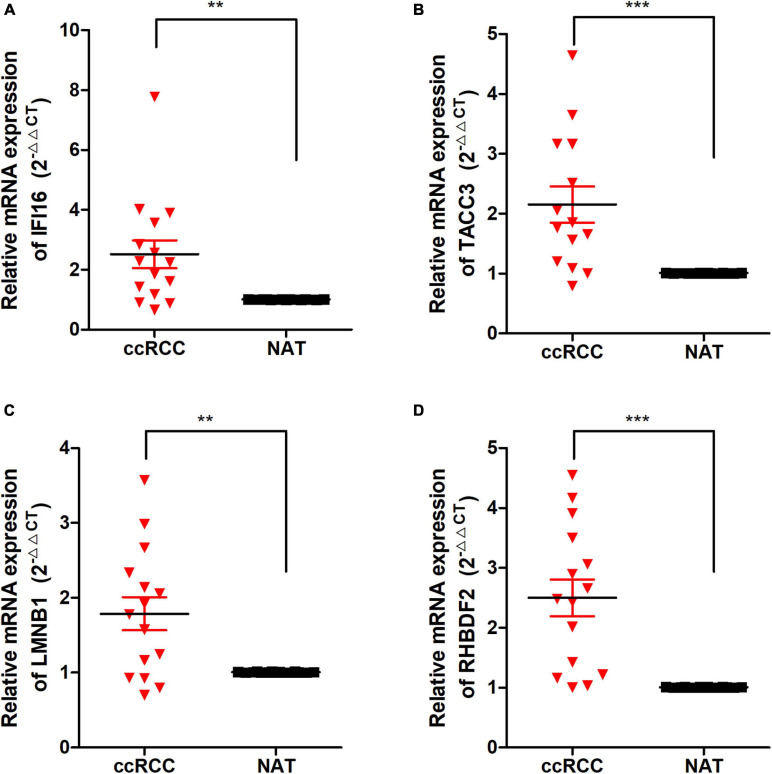
The expression of these Hub genes in human ccRCC specimens and adjacent normal tissues (ANT). **(A–D)** q-RTPCR analysis of IFI16 **(A)**, TACC3 **(B)**, LMNB1 **(C)**, and RHBDF2 **(D)** in paired ccRCC tissues (*n* = 15). GAPDH was used as a loading control. *p < 0.05, **p < 0.01, ***p < 0.001.

To detect the expression of the 4 hub genes (IFI16, LMNB1, RHBDF2, and TACC3) in ccRCC, we performed the qRT-PCR analysis in clinical specimens. The clinicopathological information of 15 ccRCC patients is shown in Supplementary Table 2. The mRNA expression of all the hub genes was significantly higher in ccRCC tissues when compared with adjacent normal tissues (Figure 9). This is consistent with the results of our bioinformatics analysis. These findings suggested that the expression of the hub genes may act as a promising biomarker for ccRCC. However, we don’t found closely correlation between the 4 signatures and the immune cells biomarkers (CD4 and CD8) in ccRCC sample (Supplementary Figure 7).

**FIGURE 10:**
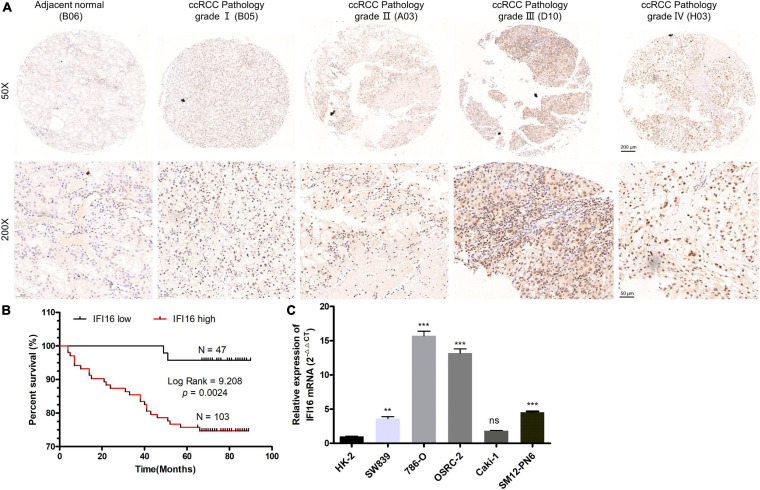
IFI16 expression profiles in ccRCC tissues and cell lines. **(A)** Representative images of IFI16 protein immunochemistry in unpaired and paired ccRCC tissues compared with adjacent normal kidney tissues. Magnification: ×50, ×200; **(B)** The Kaplan-Meier overall survival curve of ccRCC patients (*n* = 150) according to IFI16 protein expression (*p* = 0.0024, by the log-rank test). **(C)** Expression level of IFI16 in ccRCC cell lines were screened by qRT-PCR. (ns no significant; **P < 0.01; ***P < 0.001).

To further confirm the correlation of hub genes expression with tumor progression in ccRCC. We performed immunohistochemical staining (IHC) in human tissue samples to detected the protein expression of IFI16, RHBDF2, TACC3. Patients’ characteristics were retrospectively collected from the review of medical records and detailed in Supplementary Table 3. Results demonstrated that IFI16 was increased significantly in most of the paired ccRCC tissues compared with adjacent normal kidney tissues (Figure 10A and Table 3, *p* < 0.0001). However, RHBDF2 was decreased in the paired ccRCC tissues than adjacent normal kidney tissues (Supplementary Figure 8 and Supplementary Table 4, *p* < 0.0001) and TACC3 was negative in most tissues (Supplementary Figure 8 and Supplementary Table 5). Subsequently, we analyzed the clinical correlation of IFI16 and clinicopathological characteristics. As shown in Table 3, upregulation of IFI16 was significantly associated with pathology grade (*p* < 0.0001). However, no significant correlation was found between IFI16 protein expression with other clinical features. Kaplan–Meier analysis for 150 patients with follow-up data suggested that patients with higher levels of IFI16 presented significantly lower overall survival rates than those with low levels of IFI16 expression (Figure 10B, Log rank, *p* = 0.046). Furthermore, the multivariate analysis of the Cox regression model, IFI16 expression (*p* = 0.0226, HR = 5.474) was confirmed to be independent prognosis factors for ccRCC patients (Table 4). To further investigate the expression profile of IFI16 in human ccRCC, we detected IFI16 expression in four human ccRCC cell lines (i.e., SW839, OSRC-2, SM12-PN6, Caki-1, and 786-O) and found that the mRNA levels of IFI16 was relatively higher in most of the ccRCC cells (Figure 10C). These results suggested that IFI16 may be a probable independent predictor in patients with ccRCC.

**TABLE 3:** The correlation between IFI16 expression and clinicopathological characteristics was analyzed in ccRCC by IHC (*n* = 150).

Variables	Total number	IFI16		χ^2^	*p*-value ^*b*^
		High expression (+ + / + + +, n,%)	Low expression (−/ +, n,%)		
Adjacent Normal	30	2 (6.7)	28 (93.3)	12.273	< 0.0001
ccRCC	30	14 (46.7)	16 (53.3)		
**Age(years)**					
≤ 57**^*a*^**	76	53 (69.7)	23 (65.0)	0.082	0.775
> 57	74	50 (67.6)	24 (75.9)		
**Gender**					
Male	107	72 (67.3)	35 (32.7)	0.329	0.566
Female	43	31 (72.1)	12 (27.9)		
**Pathology grade**					
I	19	1 (5.3)	18 (94.7)	45.366	< 0.0001
II	94	68 (72.3)	26 (27.7)		
III- IV	37	34 (91.9)	3 (8.1)		
**T stage**					
T1a-T1b	118	78 (66.1)	40 (33.9)	2.057	0.358
T2a-T2b	21	17 (81.0)	4 (19.0)		
T3-T4	11	8 (72.7)	3 (27.3)		
**Tumor size**					
≤ 107**^*c*^**	103	68 (66.0)	35 (34.0)	0.265	0.607
≥ 107	47	35 (74.5)	12 (25.5)		
**AJCC clinical stage**					
I	119	79 (66.4)	40 (33.6)	1.521	0.467
II	19	15 (78.9)	4 (21.1)		
III- IV	12	9 (75.0)	3 (25.0)		

*^a^, mean age.*

*^b^, p-value is from χ^2^-test -test. −/ +, total expression score 0-2; + + / + + +, total expression score 3−12.*

*^c^, mean tumor size; ccRCC, Clear Cell Renal Cell Carcinoma.*

**TABLE 4:** Univariate and multivariate analysis of different prognostic parameters in patients with ccRCC by Cox regression analysis.

Covariates clinical characteristics	Univariate analysis			Multivariate analysis		
	*p*	HR	95.0% CI for HR	*p*	HR	95.0% CI for HR
Sex	0.354	0.652	0.265−1.609	0.535	0.729	0.269−1.976
Age	0.466	1.012	0.979−1.047	0.922	0.998	0.962−1.036
Pathology Grade	< 0.0001	5.030	2.486−10.178	0.088	2.151	0.892−5.188
AJCC clinical stage	< 0.0001	3.206	2.104−4.886	0.142	6.441	0.536−77.359
T stage	< 0.0001	3.091	2.013−4.747	0.441	0.367	0.029−4.701
Tumor size	< 0.0001	1.003	1.002−1.004	0.034	1.001	1.000−1.003
IFI16	0.009	6.795	1.612−28.639	0.026	5.474	1.220−24.561

*Abbreviations: CI, confidence interval; HR: Hazard Ratio.*

## Discussion

Clear cell renal cell carcinoma is a complex and highly heterogeneous disease whose pathogenesis remains unclear (Hsieh et al., 2017). Therefore, understanding the potential molecular mechanisms of ccRCC is crucial for better diagnosis, treatment and prognostic predictions (Linehan and Ricketts, 2019). Although previous studies have used high-throughput technologies and advanced bioinformatics to find novel biomarkers and therapeutic targets for ccRCC, there are inconsistencies among the DEGs analyzed in different studies (Barbieri et al., 2017).

In this study, 7 GEO datasets were integrated using RRA to minimize inconsistencies and identify robust DEGs. Enrichment analyses to explore potential biological functions of robust DEGs in ccRCC were also performed, after which hub genes associated with the pathogenesis of ccRCC were screened. Four hub genes were finally screened from the two phenotypes. Comprehensive bioinformatic analyses of the four hub genes showed that they were closely associated with the pathogenesis of ccRCC. Their protein levels were significantly up-regulated in ccRCC samples when compared to normal tissues. This high expression coincided with a poor OS, hence, these genes could be regarded as independent ccRCC prognostic factors. The GSEA analysis showed that these genes were closely related to immune signaling pathways. To the best of our knowledge, this is the first study combining RRA with WGCNA to explore hub genes involved in ccRCC pathogenesis.

Robust DEGs, such as AQP9 (Xu et al., 2019) and SULT (Li et al., 2019), are biomarkers of ccRCC and play a key role in its pathogenesis. Based on GO enrichment results of robust DEGs, studies have proved that small molecule catabolic processes and carboxylic acid biosynthetic processes are significantly associated with the initiation and progression of cancer (Sciacovelli and Frezza, 2016; DiNatale et al., 2020). Selvakumar et al. (2014) reported that active transmembrane transporter activity plays a key role in ccRCC, which was an enriched GO term of robust DEGs. Enrichment of robust DEGs in some KEGG pathways such as glycolysis/gluconeogenesis (Massari et al., 2015; Ciccarese et al., 2016) and PPAR signaling (Chang and Lai, 2019) also prove their relevance in ccRCC pathogenesis. Documented evidence suggests that ccRCC cells use the glycolytic pathway for energy production in the presence of oxygen, a phenomenon known as the Warburg Effect (Massari et al., 2015; Ciccarese et al., 2016). Dysregulated PPAR signaling pathway in pan-cancer results in dysregulated metabolism and is associated with immunosuppression (Chang and Lai, 2019). We noted multiple molecular mechanisms of robust DEGs that were closely associated with ccRCC pathogenesis.

Four hub genes (IFI16, LMNB1, RHBDF2, and TACC3) were identified in this study. Interferon-inducible 16 (IFI16), an innate immune sensor for DNA in cells, can recruit STING after DNA stimulation (Unterholzner et al., 2010). This interferon can also activate the STING signaling pathway that plays a key role in the immune escape, thereby promoting tumor progression (Lemos et al., 2016; He et al., 2017). Cai et al. reported that IFI16 promotes the progression of cervical cancer by up-regulating PD-L1 in TME through the STING-TBK1-NF-kB pathway (Cai et al., 2020). Lamin B1 (LMNB1) is associated with various cellular physiological activities, including nuclear autophagy, DNA replication and transcription, nuclear migration, DNA repair pathways, etc. (Barascu et al., 2012; Yang et al., 2019). Previous studies showed that LMNB1 was overexpressed in pancreatic cancer, liver cancer and prostate cancer (Butin-Israeli et al., 2012; Irianto et al., 2016) and its overexpression was associated with poor clinical outcomes in the cervical (Yang et al., 2019) and colon (Izdebska et al., 2018) cancers. These findings were consistent with our results. Rhomboid 5 homolog 2 (RHBDF2) induces gastric cancer cell invasiveness by regulating Transforming Growth Factor Beta 1 (TGFB1) signaling (Ishimoto et al., 2017), a finding that corresponds with our results. Mutations in RHBDF2 accelerate tumorigenesis by activating epidermal growth factor receptor (EGFR) signaling (Hosur et al., 2014) and are associated with tylosis esophageal cancer (Blaydon et al., 2012). Transforming acidic coiled-coil protein 3 (TACC3) is overexpressed in RCC cells and can promote proliferation, invasion and migration of RCC cells (Guo and Liu, 2018). Overexpression of TACC3 is associated with poor prognosis in the breast (Song et al., 2018), prostate (Qie et al., 2020) and colorectal (Du et al., 2016) cancers. Commonly occurring gene fusions such as FGFR3-TACC3 fusions are potent oncogenes that rely on mitochondrial respiration (Frattini et al., 2018). This finding is similar to the finding in our study. However, there is no documented evidence on the roles of the four hub genes (IFI16, LMNB1, RHBDF2, and TACC3) in ccRCC pathogenesis. Furthermore, we performed the qRT-PCR analysis in clinical samples and found that the mRNA expression of the four hub genes was significantly higher in ccRCC tissues when compared with adjacent normal tissues. This study highlights the roles of the four hub genes in ccRCC; however, more *in vivo* and *in vitro* experiments are needed to authenticate these findings.

Clear cell renal cell carcinoma is a highly immune-infiltrated tumor and its pathogenesis was closely associated with TME (Galon and Bruni, 2019). Immunotherapy plus targeted therapy are the new conventional approaches for systemic treatment of metastatic ccRCC (Rini et al., 2019). Our study shows that the four hub genes are involved in multiple immune-related signaling pathways and they positively correlate with Estimate scores. This shows that they were overexpressed in the TME. We hypothesized that the effect of these genes on tumor prognosis is related to tumor immunity. Based on this aspect, we found that these genes were positively correlated with multiple TILs, especially Treg cells, activated CD4 + memory T cells and CD8 + T cells. Multiple pieces of research documented that the high abundance of CD4 + T cells and CD8 + T cells in ccRCC were related to its pathogenesis and poor prognosis (Nakano et al., 2001; Remark et al., 2013). Increased Treg cell numbers can suppress anti-tumor immune responses and are correlated with poor ccRCC prognosis (Liotta et al., 2011; Kang et al., 2013; Polimeno et al., 2013). There was a negative association between resting NK and activated NK cells and the four hub genes. Low NK-cell densities were related to a worse prognosis in ccRCC (Remark et al., 2013). These results reveal that TACC3, RHBDF2, LMNB1 and IFI16 may promote tumor progression by regulating TILs in ccRCC. However, exploring the molecular mechanisms by which these genes regulate TILs in ccRCC will be significant.

Among the ICIs, cytotoxic T-lymphocyte-associated antigen 4 (CTLA4), the godfather of checkpoint inhibitors, can dampen early activation and differentiation of T cells and actively send inhibitory signals to T cells (Pardoll, 2012; Buchbinder and Desai, 2016). Programmed cell death protein 1 (PD1; also known as PDCD1) is highly expressed on Treg cells of various cancers, thereby suppressing T cell effector functions (Pardoll, 2012; Hellmann et al., 2016). Lymphocyte activation gene 3 (LAG3) and T cell membrane protein 3 (TIM3; also known as HAVCR2) have been linked to the inhibition of lymphocyte activity (Hellmann et al., 2016; Du et al., 2017). Blocking these receptors has been shown to strengthen anti-tumor immunity in tumor animal models (Pardoll, 2012). TIGIT, T cell immunoglobulin and ITIM domain, is an inhibitory immunoreceptor and an interesting cancer immunotherapy target (Manieri et al., 2017). We report that IFI16, LMNB1, RHBDF2 and TACC3 expression was positively associated with ICIs. A high expression of these genes coincided with poor ccRCC prognosis, as they likely promote ccRCC progression by tumor immune escape.

Our study provides new insights into immunotherapy and TME in ccRCC. However, there were some limitations in this study. First, retrospective study designs induce heterogeneity in results, thus, more *in vivo* and *in vitro* experiments should be performed to validate our findings. Second, the biological mechanisms of TACC3, LMNB1, RHBDF2, and IFI16 identified in this study warrant further investigation.

In conclusion, there are robust DEGs and several gene modules that are associated with the clinical pathogenesis of ccRCC. Four hub genes (IFI16, LMNB1, RHBDF2 and TACC3) were up-regulated in ccRCC tissues and correlated with ccRCC progression. These genes were associated with poor prognosis of ccRCC and may play key roles in the TME of ccRCC by regulating TILs or ICIs. However, *in vivo* and *in vitro* experiments are needed to validate the contribution of these genes in the pathogenesis of ccRCC. Additionally, more studies should be conducted to understand the molecular mechanisms of IFI16, LMNB1, RHBDF2 and TACC3 in the pathogenesis of ccRCC.

## Data Availability Statement

The original contributions presented in the study are included in the article/Supplementary Material, further inquiries can be directed to the corresponding author/s.

## Ethics Statement

The studies involving human participants were reviewed and approved by First Affiliated Hospital of Guangzhou Medical University. The patients/participants provided their written informed consent to participate in this study.

## Author Contributions

DL, XW, MW, WC, and SY performed data analyses. DL performed the experiments. MW, WC, and SY assisted in data collection. XW and DL wrote the manuscript. Funding was obtained by GZ, YL, and DL. GZ, YL, and DG supervised the study and revised the manuscript. All authors participated in preparing the manuscript and approved the final version of the manuscript.

## Conflict of Interest

The authors declare that the research was conducted in the absence of any commercial or financial relationships that could be construed as a potential conflict of interest.

## Footnotes

1 https://www.ncbi.nlm.nih.gov/geo/

2 https://cancergenome.nih.gov/

3 https://bioconductor.org/packages/release/bioc/html/limma.html

4 https://www.icesi.edu.co/CRAN/web/packages/RobustRankAggreg/

5 http://www.bioconductor.org/packages/release/bioc/html/edgeR.html

6 http://www.bioconductor.org/packages/release/bioc/html/clusterProfiler.html

7 https://cistrome.shinyapps.io/timer/

8 https://cran.microsoft.com/snapshot/2017-07-15/web/packages/pROC/index.html

9 https://cran.r-project.org/web/packages/ggstatsplot/index.html

10 https://proteomics.cancer.gov/programs/cptac

11 https://bioinformatics.mdanderson.org/estimate/index.html

12 https://cibersort.stanford.edu/

13 http://cis.hku.hk/TISIDB/index.php

14 https://cran.r-project.org/web/packages/survminer/index.html

15 https://cran.r-project.org/web/packages/survival/index.html

16 https://www.r-project.org/

## Supplementary Material

Supplementary Figure 1 Identification of differentially expressed genes. **(A)** The expression matrix of 841 robust DEGs in 72 pairs of ccRCC and adjacent normal tissues followed by unsupervised hierarchical clustering in TCGA database. **(B)** Heatmap presenting the top 20 upregulated (Red) and top 20 downregulated (Green) robust DEGs according to *p*-value. The numbers in the heatmap represent log2- fold change in each dataset calculated by the “limma” R package. DEG, differentially expressed gene.

Supplementary Figure 2 Differences in the four hub gene expressions between pan-cancer and adjacent normal tissues. **(A)** IFI16; **(B)** LMNB1; **(C)** RHBDF2; **(D)** TACC3.

Supplementary Figure 3 ROC curve analysis of IFI16, LMNB1, RHBDF2 and TACC3 **(A)** and corresponding proteins **(B)** for the diagnosis of ccRCC in the TCGA database.

Supplementary Figure 4 Positive correlations between IFI16, LMNB1, RHBDF2 and TACC3 expression (FPKM) and immune, stromal, and estimate scores in the TCGA database.

Supplementary Figure 5 Association between the expression of IFI16 **(A)**, LMNB1 **(B)**, RHBDF2 **(C)**, and TACC3 **(D)** with tumor-infiltrating lymphocytes in ccRCC. *p* < 0.05 is regarded as statistically significant. Each dot represents a ccRCC sample in the TCGA database.

Supplementary Figure 6 Kaplan-Meier survival analysis showing the higher expressions of IFI16 **(A)**, RHBDF2 **(B)**, LMNB1 **(C)**, and TACC3 **(D)** that were correlated with poor survival of ccRCC patients in the CPTAC cohort.

Supplementary Figure 7 The correlation of 4 signatures and the immune cells biomarkers (CD4 and CD8) in ccRCC samples was detected by RT -qPCR.

Supplementary Figure 8 RHBDF2 and TACC3 protein expression profiles in ccRCC tissues. **(A,B)** Representative images of RHBDF2 and TACC3 protein immunochemistry in unpaired and paired ccRCC tissues compared with adjacent normal kidney tissues. Magnification: ×50, ×200.
