# Supplementary figures and images for: Functional Assessment of Four Novel Immune-Related Biomarkers in the Pathogenesis of Clear Cell Renal Cell Carcinoma
Source: Front Cell Dev Biol. 2021 Mar 16;9:621618. doi: 10.3389/fcell.2021.621618 (PMC8007883; doi:10.3389/fcell.2021.621618)

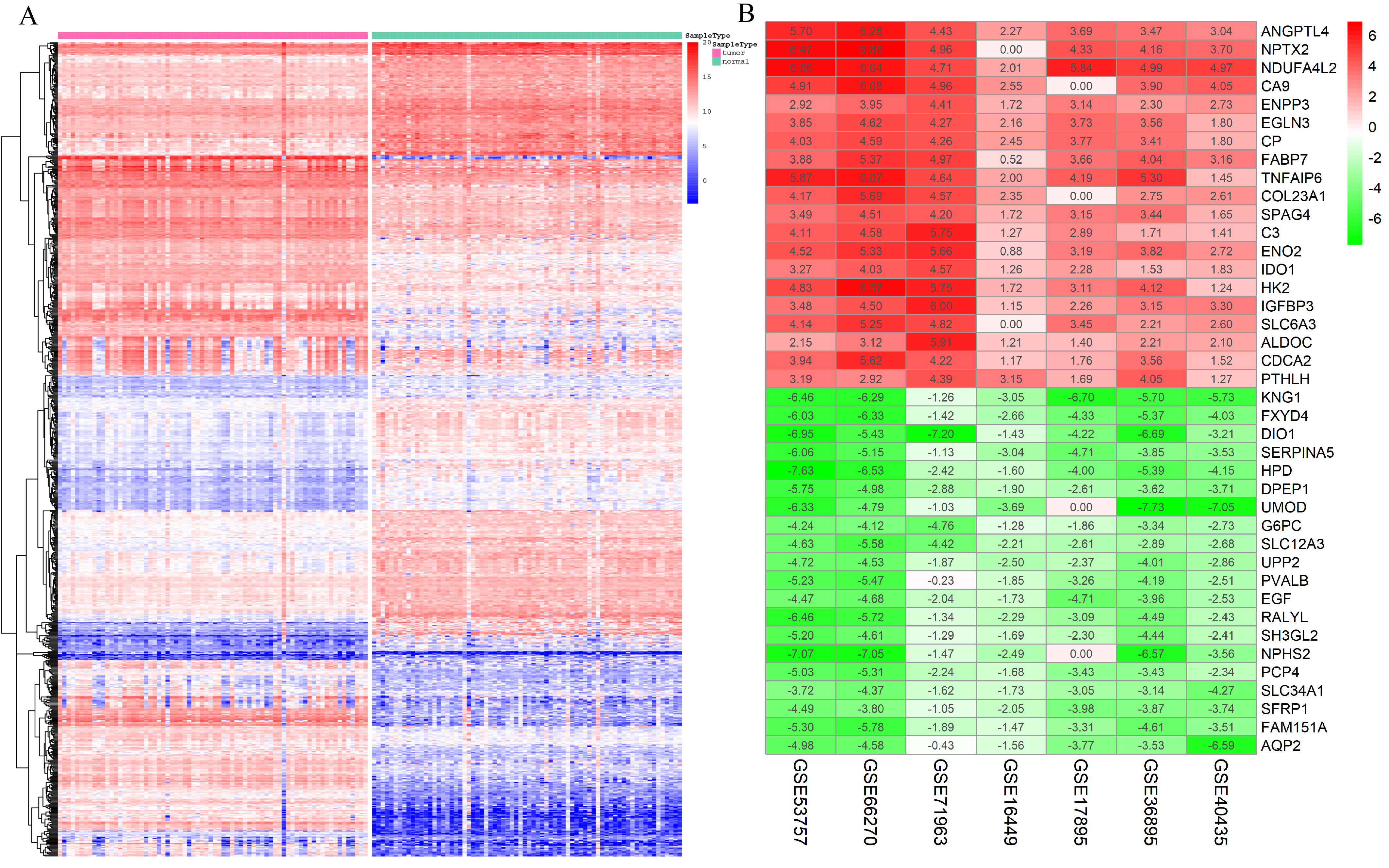

Supplement: Supplementary Figure 1 — Identification of differentially expressed genes. (A) The expression matrix of 841 robust DEGs in 72 pairs of ccRCC and adjacent normal tissues followed by unsupervised hierarchical clustering in TCGA database. (B) Heatmap presenting the top 20 upregulated (Red) and top 20 downregulated (Green) robust DEGs according to p-value. The numbers in the heatmap represent log2- fold change in each dataset calculated by the “limma” R package. DEG, differentially expressed gene. [file Image_1.TIF]

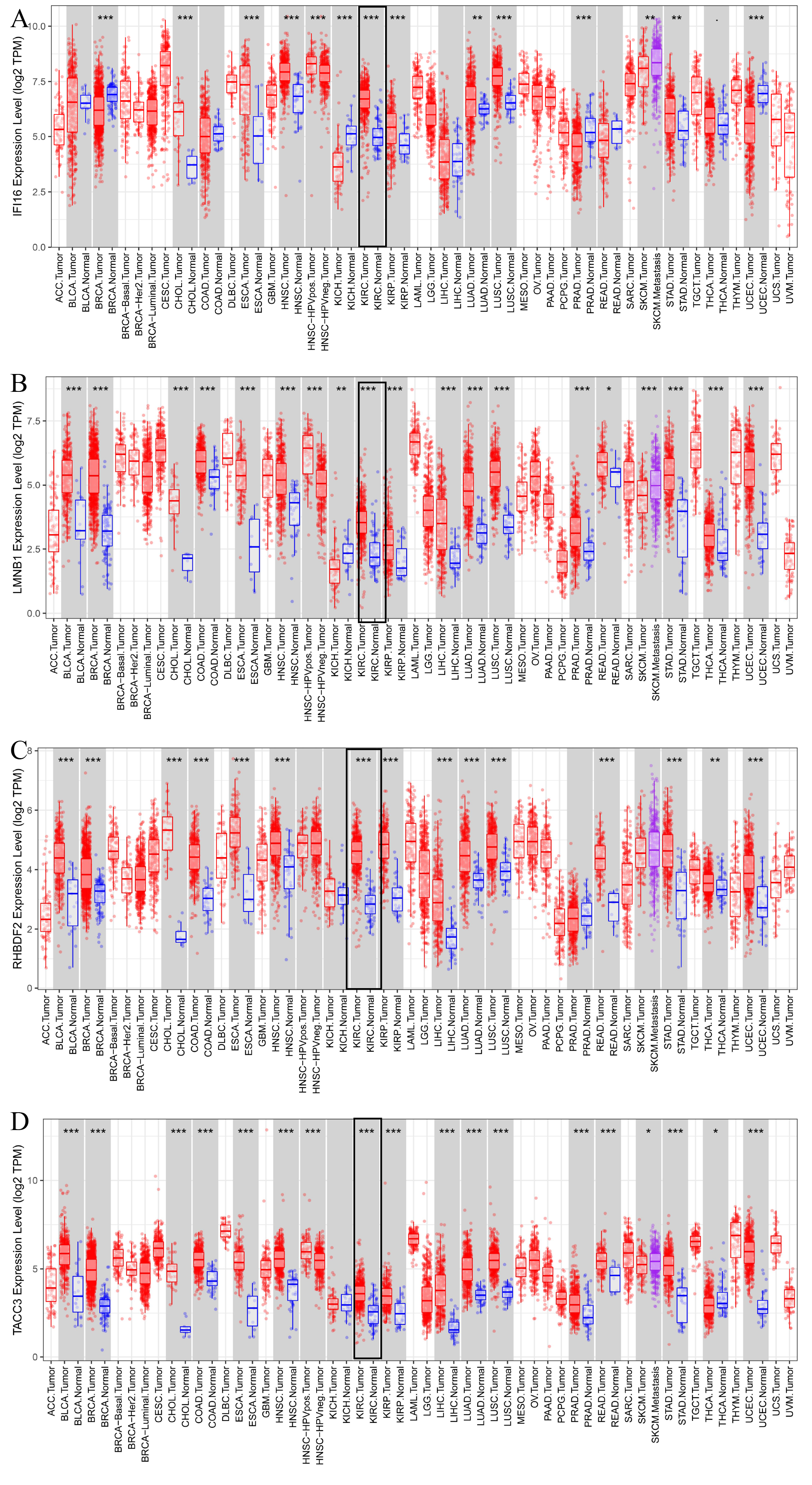

Supplement: Supplementary Figure 2 — Differences in the four hub gene expressions between pan-cancer and adjacent normal tissues. (A) IFI16; (B) LMNB1; (C) RHBDF2; (D) TACC3. [file Image_2.TIF]

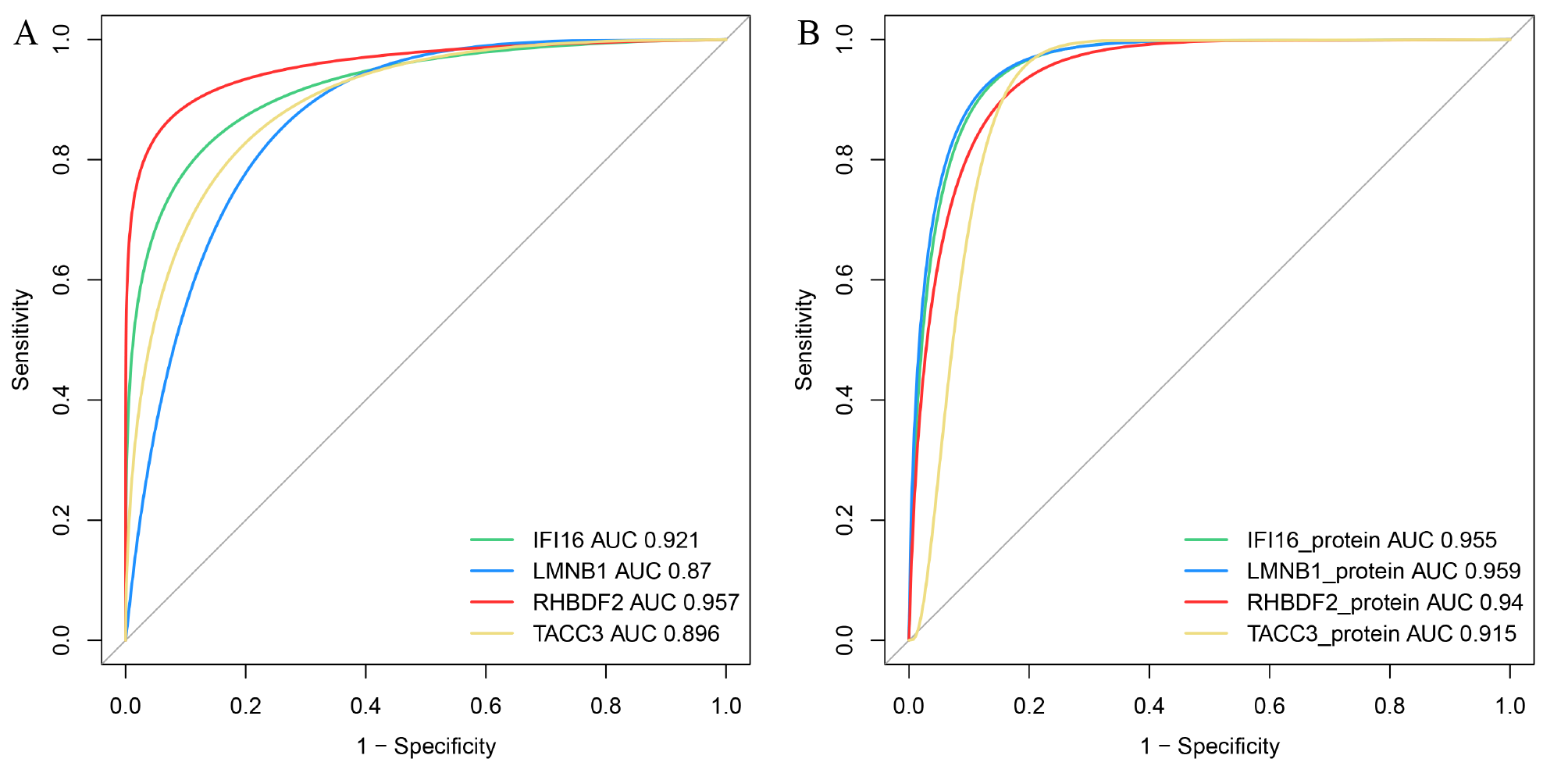

Supplement: Supplementary Figure 3 — ROC curve analysis of IFI16, LMNB1, RHBDF2 and TACC3 (A) and corresponding proteins (B) for the diagnosis of ccRCC in the TCGA database. [file Image_3.TIF]

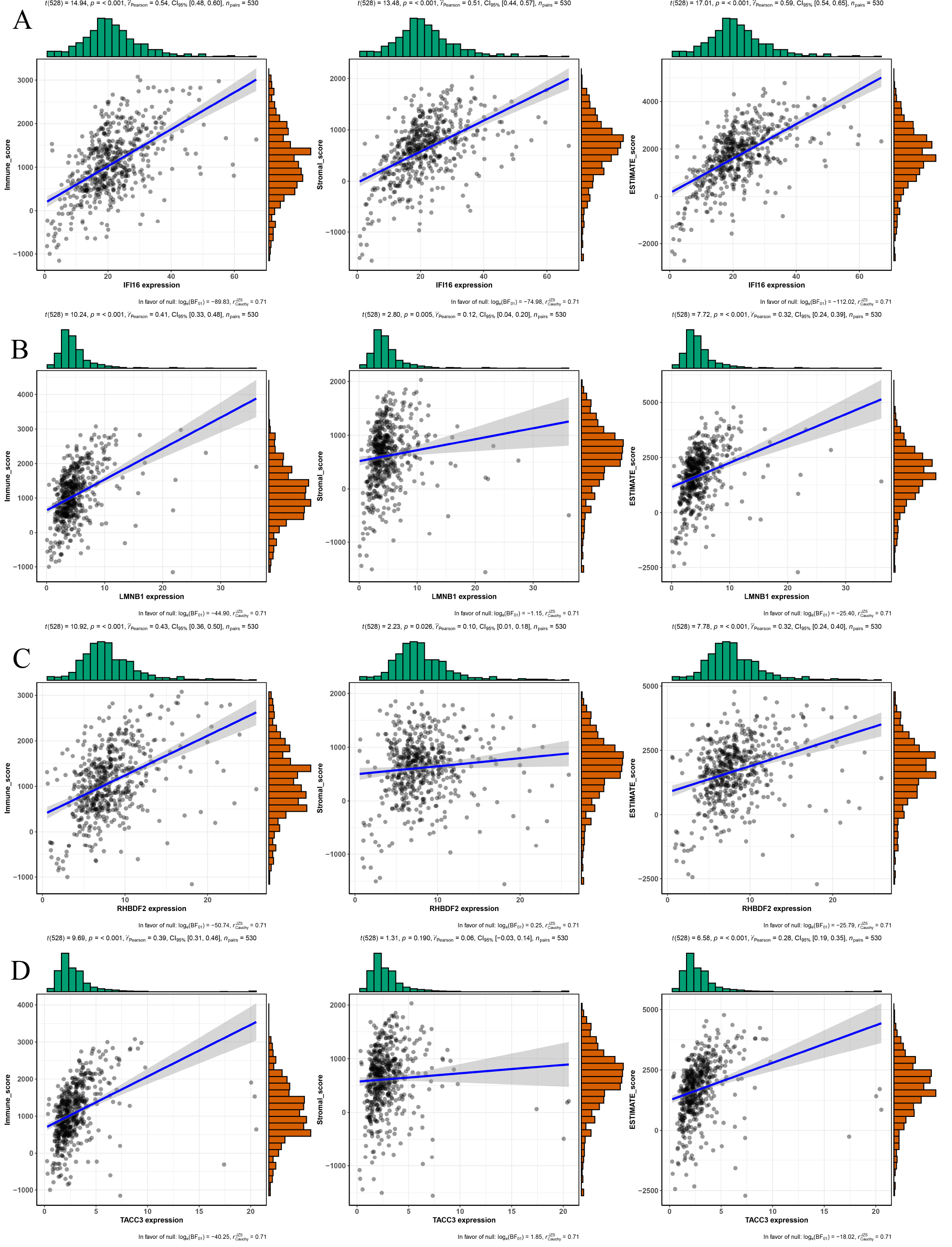

Supplement: Supplementary Figure 4 — Positive correlations between IFI16, LMNB1, RHBDF2 and TACC3 expression (FPKM) and immune, stromal, and estimate scores in the TCGA database. [file Image_4.TIF]

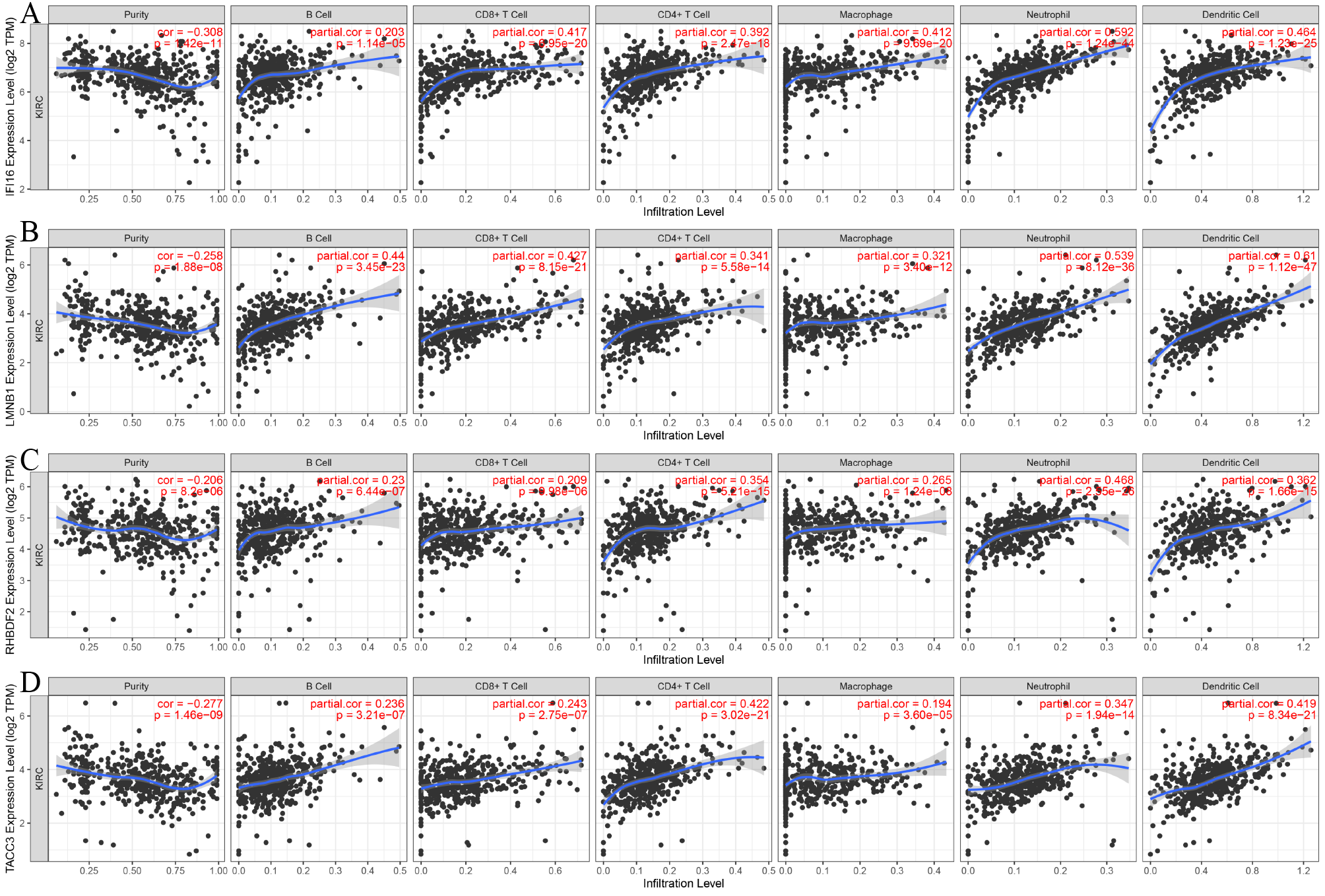

Supplement: Supplementary Figure 5 — Association between the expression of IFI16 (A), LMNB1 (B), RHBDF2 (C), and TACC3 (D) with tumor-infiltrating lymphocytes in ccRCC. p < 0.05 is regarded as statistically significant. Each dot represents a ccRCC sample in the TCGA database. [file Image_5.TIF]

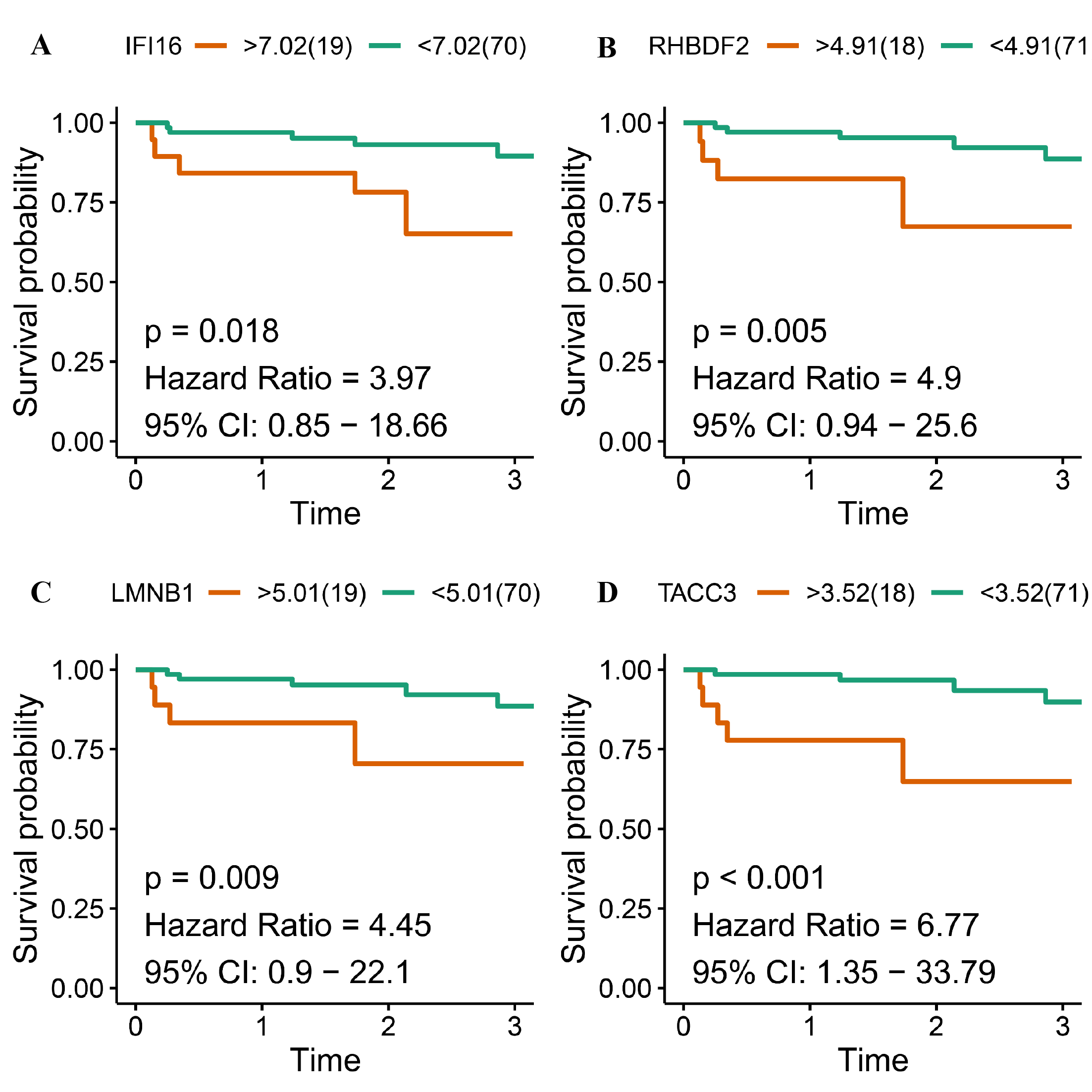

Supplement: Supplementary Figure 6 — Kaplan-Meier survival analysis showing the higher expressions of IFI16 (A), RHBDF2 (B), LMNB1 (C), and TACC3 (D) that were correlated with poor survival of ccRCC patients in the CPTAC cohort. [file Image_6.TIF]

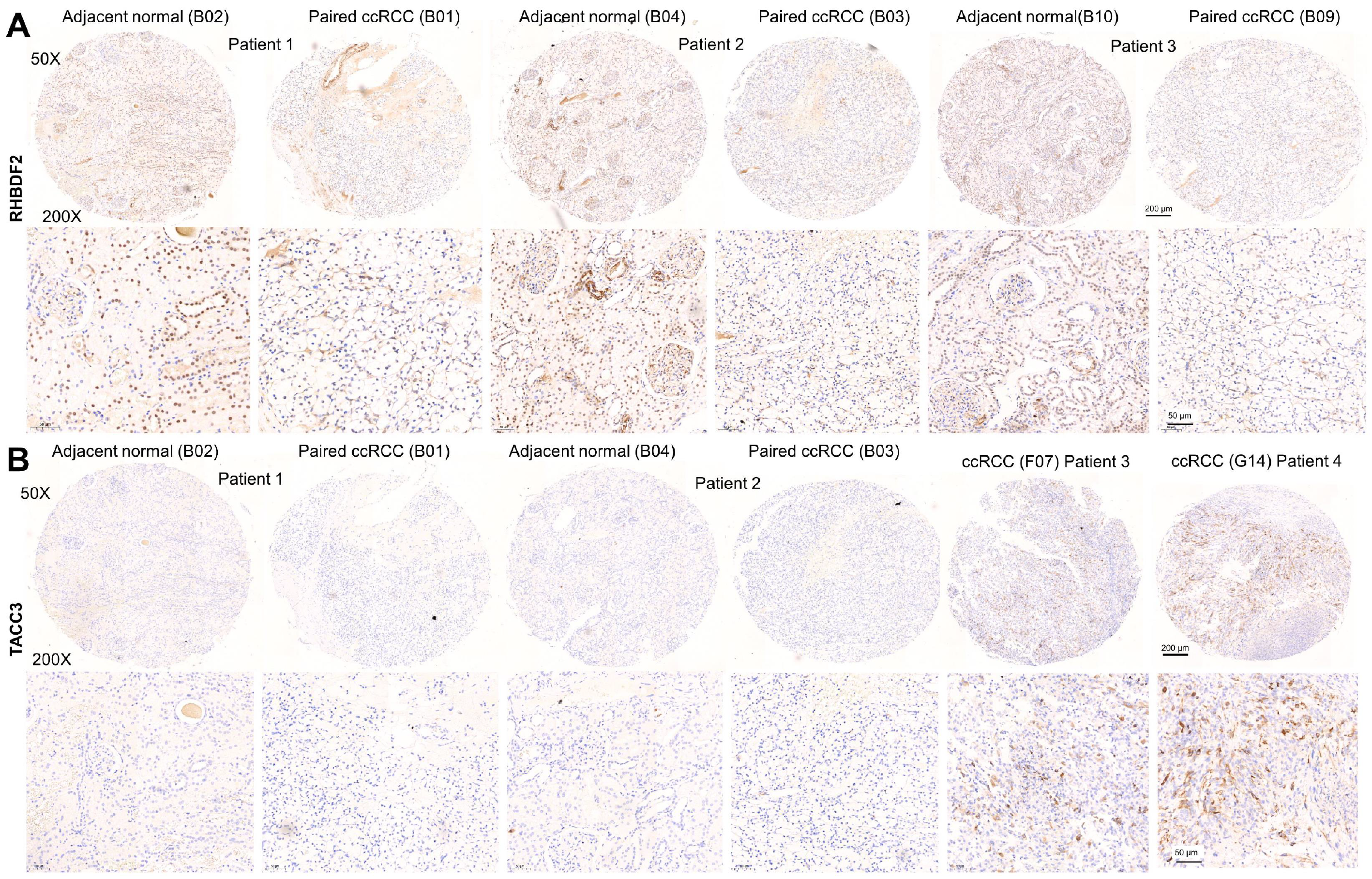

Supplement: Supplementary Figure 8 — RHBDF2 and TACC3 protein expression profiles in ccRCC tissues. (A,B) Representative images of RHBDF2 and TACC3 protein immunochemistry in unpaired and paired ccRCC tissues compared with adjacent normal kidney tissues. Magnification: ×50, ×200. [file Image_8.TIF]
