# Supplementary material for: Functional Assessment of Four Novel Immune-Related Biomarkers in the Pathogenesis of Clear Cell Renal Cell Carcinoma
Source: Front Cell Dev Biol. 2021 Mar 16;9:621618. doi: 10.3389/fcell.2021.621618 (PMC8007883; doi:10.3389/fcell.2021.621618)
Supplement: Supplementary file 12 [file Table_4.DOCX]

**Table S4. The correlation between RHBDF2 expression and clinicopathological characteristics was analyzed in ccRCC by IHC (n = 150)**

| Variables | Total Number | RHBDF2 | | | χ^2^ | *p* value **^b^** |
| --- | --- | --- | --- | --- | --- | --- |
|  |  | High expression  (++/+++, n, %) | | Low expression  (-/+, n, %) |  |  |
| Adjacent Normal | 30 | 20 (66.7) | | 10 (33.3) | **15.429** | **＜ 0.0001** |
| ccRCC | 30 | 5 (16.7) | | 25 (83.3) |  |  |
| Age (years) |  | |  |  |  |  |
| ≤ 57**^a^** | 76 | 36 (47.4) | | 40 (52.6) | 0.025 | 0.875 |
| > 57 | 74 | 36 (48.6) | | 38 (51.4) |  |  |
| Gender |  |  | |  |  |  |
| Male | 107 | 48 (44.9) | | 59 (55.1) | 1.475 | 0.225 |
| Female | 43 | 24 (55.8) | | 19 (44.2) |  |  |
| Pathology grade |  |  | |  |  |  |
| I | 19 | 3 (15.8) | | 16 (84.2) | **9.079** | **0.011** |
| II | 94 | 50 (53.2) | | 44 (46.8) |  |  |
| III- IV | 37 | 19 (51.4) | | 18 (48.6) |  |  |
| T stage |  |  | |  |  |  |
| T1a-T1b | 118 | 55 (46.6) | | 63 (53.4) | 0.823 | 0.663 |
| T2a-T2b | 21 | 12 (57.1) | | 9 (42.9) |  |  |
| T3-T4 | 11 | 5 (45.5) | | 6 (54.5) |  |  |
| Tumor size |  | |  |  |  |  |
| ≤ 107**^c^** | 103 | 51 (49.5) | | 52 (50.5) | 0.302 | 0.583 |
| ≥ 107 | 47 | 21 (44.7) | | 26 (55.3) |  |  |
| AJCC clinical stage |  |  | |  |  |  |
| I | 119 | 56 (47.1) | | 63 (52.9) | 0.225 | 0.894 |
| II | 19 | 10 (52.6) | | 9 (47.4) |  |  |
| III- IV | 12 | 6 (50.0) | | 6 (50.0) |  |  |

a：mean age. b: *p* value is from χ^2^-test -test. ‑/+: total expression score 0‑2; ++/+++: total expression score 3‑12. c: mean tumor size. ccRCC: Clear Cell Renal Cell Carcinoma
