# Supplementary material for: Functional Assessment of Four Novel Immune-Related Biomarkers in the Pathogenesis of Clear Cell Renal Cell Carcinoma
Source: Front Cell Dev Biol. 2021 Mar 16;9:621618. doi: 10.3389/fcell.2021.621618 (PMC8007883; doi:10.3389/fcell.2021.621618)
Supplement: Supplementary file 13 [file Table_5.DOCX]

**Table S5. The correlation between TACC3 expression and clinicopathological characteristics was analyzed in ccRCC by IHC (n = 150)**

| Variables | Total number | | TACC3 | | χ^2^ | *p* value^b^ |
| --- | --- | --- | --- | --- | --- | --- |
|  |  |  | High expression  (++/+++, n, %) | Low expression  (-/+, n, %) |  |  |
| Adjacent Normal | 30 | | 0 (0) | 30 (100) | **-** | **ns** |
| ccRCC | 30 | | 0 (16.7) | 30 (100) |  |  |
| Age(years) |  |  |  |  |  |  |
| ≤ 57^a^ | 76 | | 3 (3.9) | 73 (96.1) | 0.001 | 0.973 |
| > 57 | 74 | | 3 (4.1) | 71 (95.9) |  |  |
| Gender |  | |  |  |  |  |
| Male | 107 | | 2 (1.9) | 105 (98.1) | **4.413** | **0.036** |
| Female | 43 | | 4 (9.3) | 39 (90.7) |  |  |
| Pathology grade |  | |  |  |  |  |
| I- II | 113 | | 1 (0.9) | 112 (99.1) | **11.576** | **0.001** |
| III- IV | 37 | | 5 (13.5) | 32 (86.5) |  |  |
| T stage |  | |  |  |  |  |
| T1-T2 | 139 | | 6 (4.3) | 133 (95.7) | 0.933 | 0.334 |
| T3-T4 | 11 | | 0 (0.0) | 11 (100) |  |  |
| Tumor size |  |  |  |  |  |  |
| ≤ 107^c^ | 103 | | 4 (3.9) | 99 (96.1) | 0.012 | 0.914 |
| ≥ 107 | 47 | | 2 (4.3) | 45 (95.7) |  |  |
| AJCC clinical stage |  | |  |  |  |  |
| I | 119 | | 4 (3.4) | 115 (96.6) | 0.604 | 0.361 |
| II - IV | 31 | | 2 (93.5) | 29 (93.5) |  |  |

a：mean age. b: *p* value is from χ^2^-test -test. ‑/+: total expression score 0‑2; ++/+++: total expression score 3‑12. c: mean tumor size. ccRCC: Clear Cell Renal Cell Carcinoma
